# S3‐Leitlinie Diagnostik und Therapie der Alopecia areata – Teil 1: Diagnostik und Epidemiologie

**DOI:** 10.1111/ddg.70065

**Published:** 2026-07-07

**Authors:** Ulrike Blume‐Peytavi, Andria Constantinou, Tsenka Tomova‐Simitchieva, Adrian Tanew, Michael Sticherling, Hermann Girschick, Annika Vogt, Uwe Schwichtenberg, Uwe Gieler, André Märtens, Kerstin Zienert, Claudia Stenders, Ricardo Niklas Werner, Doris Wilborn

**Affiliations:** ^1^ Klinik für Dermatologie Venerologie und Allergologie Charité – Universitätsmedizin Berlin Corporate Member of Freie Universität Berlin Humboldt‐Universität zu Berlin und Berlin Institute of Health Berlin Deutschland; ^2^ Privatordination Wien Österreich; ^3^ Hautklinik Universitätsklinikum Erlangen Erlangen Deutschland; ^4^ Klinik für Kinder‐ und Jugendmedizin Vivantes Netzwerk für Gesundheit GmbH Klinikum im Friedrichshain Berlin Deutschland; ^5^ Derma Nord Hautarztpraxis, Deutschland; ^6^ Psychodermatology Competence Centre Universitätsklinikum Gießen Vitos Psychosomatik Gießen Gießen Deutschland; ^7^ Friseur Berlin Deutschland; ^8^ Alopecia Areata Deutschland e.V., Deutschland; ^9^ Klinik für Dermatologie, Venerologie und Allergologie Division of Evidence Based Medicine (dEBM) Charité Universitätsmedizin Berlin Corporate Member of Freie Universität Berlin Humboldt‐Universität zu Berlin und Berlin Institute of Health Berlin Deutschland

**Keywords:** Alopecia areata, Diagnostik, Epidemiologie, Komorbidität, prognostische Faktoren, Risikofaktoren, Alopecia areata, comorbidities, diagnosis, epidemiology, prognostic factors, risk factors

## Abstract

In dem vom Innovationsausschuss beim gemeinsamen Bundesausschuss (G‐BA) geförderten Projekt wurde von 2023 bis 2025 die S3‐Leitlinie zur Diagnostik und Therapie der Alopecia areata (AA) entwickelt. Die interdisziplinäre Expertengruppe bestand aus Vertretern der Deutschen Dermatologischen Gesellschaft, insbesondere aus der AG Pädiatrische Dermatologie, des Berufsverbands der Deutschen Dermatologen, der Deutschen Gesellschaft für Psychosomatische Medizin und Psychotherapie, der Deutschen Gesellschaft für Kinder‐ und Jugendmedizin, der Österreichischen Gesellschaft für Dermatologie und Venerologie, einem Friseur als externer Berater und zwei Patientenvertreterinnen.

Im ersten von zwei Teilen stellen wir die zentralen Aussagen zur Epidemiologie und die Empfehlungen zur Diagnostik vor. Daten aus Deutschland zeigen eine populationsbasierte Prävalenz der AA von 0,22 % (95 %‐KI 0,21–0,22) für 2016 und 0,21 % (95 %‐KI 0,20–0,22) für 2020. Somit waren in Deutschland 170 000 Menschen im Jahr 2020 an einer AA erkrankt. Internationale Daten zeigen eine gepoolte Prävalenz von 2,1 %, allerdings mit großen regionalen Unterschieden. Daten zur Inzidenz der AA aus Deutschland zeigen, dass im Jahr 2020 bei 72 Patienten pro 100 000 eine AA neu diagnostiziert wurde (RR 0,072 %; 95 %‐KI 0,07–0,08). Hochgerechnet auf die deutsche Bevölkerung bedeutet dies, dass etwa 70 000 Menschen im Jahr 2020 neu an einer AA erkrankt waren.

## EINLEITUNG

In diesem ersten Teil der Publikation werden Empfehlungen und Auszüge der Hintergrundtexte der S3‐Leitlinie *Diagnostik und Therapie der Alopecia areata* zur Diagnostik inklusive Epidemiologie, Komorbidität, prognostischen Faktoren und Lebensqualität der Alopecia areata (AA) vor Therapie vorgestellt. Dieser erste Teil basiert auf der Langversion vom 18.09.2025. Sie wurde gemäß dem Regelwerk der AWMF Version 2.0 von 2020,^1^ und der Version 2.1 (2023),^2^ von Januar 2023 bis Oktober 2025 entwickelt. Der zweite Teil enthält die Empfehlungen und Hintergrundtexte der Kapitel Therapie, Lebensqualität im Verlauf der Therapie, psychosoziale Unterstützung und kosmetische Angebote und sollte gemeinsam mit dem 1. Teil der Leitlinie zu Diagnostik und Epidemiologie genutzt werden. Patientenzielgruppe der Leitlinie sind Patienten mit einer AA in allen Altersgruppen, die Empfehlungen wurden spezifisch für Kinder, Jugendliche und Erwachsene formuliert.

Die AA vom Typ der Alopecia areata circumscripta (AC) als häufigste Erstmanifestation einer AA und vom seltenen Typ der Alopecia areata totalis (AT, ORPHA: 700), und der Alopecia areata universalis (AU, ORPHA:701) tritt häufig bereits im Kindes‐ und Jugendalter auf und kann akut oder auch chronisch rezidivierend bis hin zu komplettem Verlust der Kopf‐, Gesichts‐ und Körperhaare führen. Der sichtbare Haarverlust führt neben funktionellen Einschränkungen (Augenentzündungen, erhöhte UV‐Exposition) oftmals zu einer nicht zu unterschätzenden enormen emotionalen und psychosozialen Belastung. Es kommt häufig zu sozialer Stigmatisierung, Mobbing, bis hin zu sozialem Rückzug und nachhaltiger Einschränkung der Lebensqualität. Sechsundsechzig Prozent aller AA‐Patienten erfahren die Erstmanifestation ihrer Erkrankung im Kindes‐ und Jugendalter bis zum Ende des dritten Lebensjahrzehnts. Insbesondere sind in der prägenden Sozialisierungsphase Kinder, Jugendliche und junge Erwachsene in ihrer familiären und Berufsfindungsphase häufig nachhaltig beeinträchtigt. Evidenzbasierte diagnostische und therapeutische Empfehlungen für die verschiedenen Formen der AA fehlten allgemein in Deutschland, sind aber speziell für diese Altersgruppe mit besonderen Bedürfnissen dringend erforderlich.

Das dieser Veröffentlichung zugrundliegende Projekt wurde mit Mitteln des Innovationsausschusses beim Gemeinsamen Bundesausschuss (G‐BA) unter dem Förderkennzeichen 01VSF22016 (S3‐LL AA) im Zeitraum vom 01. Januar 2023 bis zum 30. Juni 2025 gefördert.

## METHODE

Die methodischen Schritte der Leitlinienentwicklung wurden ausführlich im Leitlinienreport beschrieben (siehe auch Online‐Supplement und Webseite der AWMF https://register.awmf.org/de/leitlinien/detail/013‐104). Kurz zusammengefasst: Die Leitliniengruppe verabschiedete im Kick‐off‐Treffen im Februar 2023 die 45 Schlüsselfragen, die der systematischen Literatursuche zugrunde lagen. Dabei wurde festgelegt, dass der Schwerpunkt der Literatursuche sich auf epidemiologische und therapeutische Fragestellungen konzentrieren sollte. Basierend auf den Ergebnissen der Literatursuche, ‐bewertung und ‐zusammenfassung konnte die Leitliniengruppe 79 Empfehlungen für die gesamte Leitlinie in der Konsensuskonferenz im September 2024 verabschieden.

### Konsensusprozess

Als Grundlage zur einheitlichen Formulierung der Empfehlungen nutzte die Leitliniengruppe das Rahmenwerk der *Grading of Recommendations Assessment, Development and Evaluation (GRADE) Working Group* (Tabelle 1).^3^

**TABELLE 1:** Schema zur Graduierung von Empfehlungen.

Empfehlungsstärke	Ausdrucksweise	Symbol	Interpretation
Starke Empfehlung für eine Vorgehensweise	Soll	**↑↑**	Wir sind der Auffassung, dass alle oder fast alle informierten Menschen eine Entscheidung zugunsten dieser Intervention treffen würden. Kliniker müssen sich weniger Zeit für den Prozess der Entscheidungsfindung mit Patienten nehmen und können diese Zeit stattdessen für die Überwindung von Barrieren bei der Implementierung und der Therapieadhärenz einsetzen. In den meisten klinischen Situationen kann die Empfehlung als allgemeine Vorgehensweise übernommen werden.
Schwache Empfehlung für eine Vorgehensweise	Sollte	**↑**	Wir sind der Auffassung, dass die meisten informierten Menschen, ein substanzieller Anteil jedoch nicht, eine Entscheidung zugunsten dieser Intervention treffen würden. Kliniker und andere Anbieter von Gesundheitsleistungen müssen sich mehr Zeit für den Prozess der Entscheidungsfindung mit Patienten nehmen. Entscheidungsprozesse im Gesundheitssystem erfordern eine tiefgehende Diskussion und die Einbeziehung vieler Interessengruppen.
Empfehlung offen/keine Empfehlung bezüglich einer Vorgehensweise	Kann erwogen/verzichtet werden	0	Zurzeit kann eine Empfehlung für oder gegen diese Intervention aufgrund bestimmter Gegebenheiten nicht getroffen werden (zum Beispiel unklares oder ausgeglichenes Nutzen‐Risiko‐Verhältnis, keine verfügbare Evidenz)
Schwache Empfehlung gegen eine Vorgehensweise	Sollte nicht	**↓**	Wir sind der Auffassung, dass die meisten informierten Menschen, ein substanzieller Anteil jedoch nicht, eine Entscheidung gegen diese Intervention treffen würden.
Starke Empfehlung gegen eine Vorgehensweise	Soll nicht	**↓↓**	Wir sind der Auffassung, dass alle oder fast alle informierten Menschen eine Entscheidung gegen diese Intervention treffen würden. In den meisten klinischen Situationen kann die Empfehlung als allgemeine Vorgehensweise übernommen werden.

*Wording in den Empfehlungen*: Zur einfachen Erkennbarkeit der Empfehlungen wurden diese in separate Kästchen eingefügt, die einem einheitlichen Aufbau folgten: In der linken Spalte erscheint die laufende Nummer der Empfehlung. Die mittlere Spalte zeigt den Empfehlungstext unter Verwendung der standardisierten Begriffe. Darunter erscheint die Stärke des Konsenses in der Leitlinienkommission und die Evidenzbasis inklusive Literaturangaben (konsensbasiert/evidenzbasiert). In der rechten Spalte wird durch Pfeile und eine farbliche Hinterlegung die Richtung und die Stärke der Empfehlung gekennzeichnet. Die Klassifikation der Konsensstärke findet sich in Tabelle 2.

**TABELLE 2:** Klassifikation der Konsensusstärke.

Klassifikation der Konsensusstärke	
Starker Konsens	> 95 % der Stimmberechtigten
Konsens	> 75–95 % der Stimmberechtigten
Mehrheitliche Zustimmung	> 50–75 % der Stimmberechtigten
Keine mehrheitliche Zustimmung	< 50 % der Stimmberechtigten

Alle Mitglieder der Leitliniengruppe erhielten den vollständigen Hintergrundtext zur Vorbereitung auf die Vorab‐Online‐Abstimmung der Empfehlungen. Das Delphi‐Verfahren bot die Möglichkeit der Abstimmung und auch Kommentierung der vorgelegten Empfehlungsentwürfe. Die Empfehlungen mit mehr als 95 % Zustimmung wurden in der Konsensuskonferenz vorgelesen und galten als abgestimmt. Die noch offenen Empfehlungen wurden diskutiert und final abgestimmt. Moderiert wurde die Konferenz, die als hybride Veranstaltung stattfand, von Herrn Priv.‐Doz. Dr. Ricardo Werner.

### Externer Review/Freigabe durch die Fachgesellschaften/Implementierung

Die Langversion, die Patientenversion und die Algorithmen der Leitlinie wurden der Deutschen Dermatologischen Gesellschaft (DDG) als federführende Fachgesellschaft und den weiteren beteiligten Fachgesellschaften und Organisationen zur Kommentierung und Zustimmung vorgelegt. Alle Rückmeldungen und deren Umgang damit wurden im Leitlinienreport festgehalten. Die Implementierung erfolgt durch die Veröffentlichung auf den Webseiten der AWMF, der DDG und den weiteren beteiligten Fachgesellschaften und des Alopecia Areata e.V.

### Aktualisierung und Gültigkeit

Die Leitlinie hat eine Gültigkeit von fünf Jahren (bis zum 17.09.2030) und soll nach diesem Zeitraum bei Änderungen in zentralen Themen aktualisiert werden.

## DEFINITION DER ALOPECIA AREATA

Die Alopecia areata ist eine chronische, immunvermittelte Krankheit, die durch einen akut einsetzenden, nicht vernarbenden Haarausfall gekennzeichnet ist, der von kleinen, umschriebenen haarlosen Arealen auf der Kopfhaut bis zum vollständigen Verlust der Kopf‐ und Körperhaare reicht. Nach heutigem Kenntnisstand geht man davon aus, dass der Zusammenbruch des Immunprivilegs (IP) des Haarfollikels (HF) zusammen mit genetischen und äußeren Faktoren für die Manifestation der Erkrankung hauptverantwortlich ist.^4^

Die AA kann mit einer erheblichen Einschränkung der Lebensqualität und erheblichen psychosozialen und somatischen Begleiterkrankungen einhergehen.

**Table:** 

	**Statement**	
**S1**	Bei der AA handelt es sich um eine Autoimmunerkrankung, die mit einer erheblichen Einschränkung der Lebensqualität und erheblichen psychosozialen und somatischen Begleiterkrankungen einhergehen kann.	
	Starker Konsens	**Konsensbasiert**

### Epidemiologie

#### Prävalenz

Vorgestellt werden hier Ausschnitte aus der Langversion der Leitlinie, insbesondere die epidemiologischen Daten aus Deutschland, weitere internationale Daten sind in der Langversion zu finden.

Daten zur populationsbasierten Prävalenz der AA aus Deutschland zeigen folgende Ergebnisse. Augustin et al. 2024 haben Daten einer repräsentativen 40 %‐Stichprobe aller Erwachsenen, die zwischen 2016 und 2020 bei einer deutschen gesetzlichen Krankenkasse (DAK‐Gesundheit) versichert waren (n = 2,88 Millionen) ausgewertet. Basierend auf mindestens einer relevanten ambulanten oder stationären Diagnose der *International Classification of Diseases* (ICD)‐10 L63 (L63, L 63.0, L 63.1, L 63.2, L 63.8, L63.9) wurden die jährliche AA‐Prävalenz und ‐Inzidenz (ICD‐10 L63) und darüber hinaus die Häufigkeit von Komorbidität für 2016 bis 2020 berechnet.^5^

Sie errechneten eine populationsbasierte Prävalenz der AA von 0,22 % (95 %‐Konfidenzintervall [KI] 0,21–0,22) für 2016 und 0,21 % (95 %‐KI 0,20–0,22) für 2020. Im Jahr 2020 hatten somit 210 Personen pro 100 000 die Diagnose einer AA (alters‐ und geschlechtsstandardisierte Prävalenzrate 0,21 %). Bei einer Hochrechnung auf die deutsche Bevölkerung angewendet, würde diese Prävalenzrate bedeuten, dass 170 000 Menschen in 2020 an einer AA erkrankt waren. Die Autoren ermittelten darüber hinaus, dass die in der Auswertung eingeschlossenen Frauen ab der Altersgruppe > 40 Jahre häufiger betroffen waren als Männer (0,2 % Frauen gegenüber 0,1 % Männern). In der Altersgruppe 70 bis < 80 Jahre war die Prävalenz der Alopecia areata bei Frauen sogar mehr als 6,5‐mal so hoch wie bei Männern. In der Altersgruppe < 40 Jahre waren die Männer etwas häufiger betroffen als die Frauen. Höhere Prävalenz‐ und Inzidenzraten für AA wurden in Hessen (Prävalenz 215 pro 100 000; Inzidenz 73 pro 100 000), in Nordrhein‐Westfalen (Prävalenz 210 pro 100 000; Inzidenz 73 pro 100 000) und in Bremen (Prävalenz 235 pro 100 000; Inzidenz 62 pro 100 000) festgestellt. Aber auch in Berlin und Brandenburg waren die Prävalenzraten eher höher (beide Prävalenz 211 pro 100 000). Am niedrigsten war die Prävalenz in Thüringen, Hamburg und Mecklenburg‐Vorpommern.^5^

#### Inzidenz

Daten zur Inzidenz der AA aus Deutschland zeigen, dass im Jahr 2020 bei 72 Patienten pro 100 000 eine AA neu diagnostiziert wurde (relatives Risiko [RR] 0,072 %; 95 %‐KI 0,07–0,08). Hochgerechnet auf die deutsche Bevölkerung bedeutet dies, dass im Jahr 2020 etwa 70 000 Menschen neu an AA erkrankt waren.^5^

## RISIKOFAKTOREN

Unter Risikofaktoren in der Medizin versteht man „Vorläufer und Prädiktoren von Krankheiten sowie Gesundheitsstörungen. Ein Risikofaktor gibt Auskunft über eine potenzielle, sich direkt oder indirekt und in der Regel erst mit zeitlicher Verzögerung manifestierende Gefährdung der Gesundheit.“^6^

Im Zusammenhang mit AA werden bestimmte Erkrankungen, insbesondere weitere Autoimmunerkrankungen, gehäuft beobachtet. Einige dieser Erkrankungen gelten als Komorbidität im Sinne von zeitgleich auftretenden Begleiterkrankungen. Ob Erkrankungen explizit als Risikofaktoren für eine AA gelten, und somit von Komorbidität abgegrenzt werden können, zeigen Ergebnisse aus epidemiologischen Studien. Dafür eignen sich vor allem Fall‐Kontroll‐Studien (retrospektiv) und zweiarmige Kohortenstudien (prospektiv). Dabei wird verglichen, ob Personen mit einer bestimmten Erkrankung in der Vergangenheit häufiger eine bestimmte Exposition aufwiesen als Personen ohne diese Erkrankung. Mit den Kennziffern *Odds Ratio* (OR) oder *Hazard Ratio* (HR) wird die Bedeutung des jeweiligen Faktors rechnerisch ermittelt.

Folgende Faktoren wurden von 50 internationalen dermatologischen Experten als Risikofaktoren für eine AA konsentiert: positive Familienanamnese für AA oder Autoimmunerkrankungen, Genetik, Eigenanamnese für Autoimmunerkrankungen, Schilddrüsenerkrankungen, Vitiligo, Atopie und/oder atopische Dermatitis.^7^

Eisenmangel, Schwangerschaft sowie Impfungen haben laut Meah et al. 2020 keinen Einfluss auf die Entstehung einer AA. Umweltfaktoren, Trauma und akuter Stress werden als Triggerfaktoren für die Entstehung von AA genannt.^7^

Tabelle 3 fasst die Ergebnisse der eingeschlossenen Studien zu den Risikofaktoren zusammen. Risikofaktoren, zu denen Studienergebnisse mit einem positiven Zusammenhang gefunden wurden, als auch Studienergebnisse, die auf keinen Zusammenhang hinweisen, werden dargestellt. Zum Vitamin‐D‐Mangel liegen widersprüchliche Ergebnisse vor, sodass Vitamin‐D‐Mangel nach der derzeitigen Studienlage nicht als Risikofaktor bewertet wird. Auch zu Schilddrüsenerkrankungen ist die Evidenzlage widersprüchlich: Wenn alle Schilddrüsenerkrankungen zusammen analysiert werden, zeigt sich ein positiver Zusammenhang, Subgruppenanalysen zeigen aber auch teilweise keinen Zusammenhang, zum Beispiel für die Hypothyreose und auch die Hyperthyreose.

**TABELLE 3:** Effektschätzer zu Risikofaktoren der Alopecia areata.

Risikofaktor	Endpunkt	Positiver Zusammenhang (Referenzen)	Kein Zusammenhang (Referenzen)
Afro‐amerikanische Ethnie (Vergleich mit Weißen, “Whites“)^8^	AA	OR 1,77 (95 %‐KI 1,37‐2,28)^8^	
Asiatische Ethnie (Vergleich mit Weißen, „Whites“)^8^	AA		OR 0,40 (95 %‐KI 0,32‐0,50) ^8^
Schilddrüsenerkrankung	AA	OR 1,91 (95 %‐KI 1,23–2,94)^9^	
Hypothyreose	AA	HR 1,88 (95 %‐KI 1,30–2,71)^10^	OR 1,28 (95 %‐KI 0,98‐1,67) ^9^
Hyperthyreose	AA	aHR 9,29 (95 %‐KI 7,11–12,14)^11^	OR 1,22 (95 %‐KI 0,59‐2,52) ^9^
Morbus Basedow	AA	aHR 8,66 (95 %‐KI 6,03–12,42)^11^	
Thyreoiditis	AA	aHR 6,42 (95 %‐KI 3,15–13,11)^11^	
Hashimoto Thyreoiditis	AA		aHR 2,70 (95 %‐KI 0,75–9,70) ^11^
Vitiligo	AA	HR 3,13 (95 %‐KI 1,08–9,10)^10^	
Systemischer Lupus erythematodes	AA	HR 5,43 (95 %‐KI 2,11–13,97)^10^	
rheumatoide Arthritis	AA	HR 1,66 (95 %‐KI 1,09–2,52)^10^	
Psoriasis	AA	HR 2,01 (95 %‐KI 1,00–4,03)^10^	
Atopische Dermatitis	AA	OR 9,72 (95 %‐KI 4,38–21,59)^12^ RR 2,98 (95 %‐KI 1,36–6,53)^12^ OR 2,36 (95 %‐KI 1,52‐3,67)^13^ OR 2,79 (95 %‐KI 1,27‐6,13)^14^	
Atopische Dermatitis	AU	OR 2,98 (95 %‐KI 1,95‐4,55)^13^	
Metabolisches Syndrom: Hyperlipidämie	AA		aHR 0,71 (95 %‐KI 0,42‐1,19) ^15^
Metabolisches Syndrom: Übergewicht	AA		aHR 1,01 (95 %‐KI 0,42‐2,43) ^15^
Vitamin‐D‐Mangel	AA	OR 2,3 (95 %‐KI 2,2–3,1)^16^	HR 1,08 (95 %‐KI 0,68–1,73) ^17^
Bakterielle Infektionen	AA	OR 1,8 (95 %‐KI 1,56‐2,08)^18^	
Virale Infektionen	AA	OR 2,0 (95 %‐KI 1,67‐ 2,38)^18^	
Helicobacter‐pylori‐Infektion	AA	OR 1,57 (95 %‐KI 1,19–2,05)^19^	
Hepatitis‐C‐Infektion	AA	aHR 6,69 (95 %‐KI 4,28–10,44)^20^	
Humanes Papilloma Virus (HPV)	AA	aHR 2,55(95 %‐KI 1,88‐3,47)^21^	
Depression	AA	aHR 11,61 (95 %‐KI 9,92–13,59)^22^ aHR 1,90 (95 %‐KI 1,67‐2,15)^23^ RR 1,66 (95 %‐KI 1,24‐2,2)^24^ OR 2,71; (95 %‐KI 1,52‐4,82)^25^	

*Abk*.: AA, Alopecia areata; AU, Alopecia universalis; aHR, adjustierte *Hazard Ratio*; HR, *Hazard Ratio*; OR, *Odds Ratio*; RR, Relatives Risiko; KI, Konfidenzintervall; HPV, Humanes Papillomavirus

## ÄTIOPATHOGENESE UND FORMEN DER AA

Für die Inhalte dieses Kapitel wird auf die Langversion verwiesen.

## DIAGNOSTIK UND DIFFERENZIALDIAGNOSTIK

**ABBILDUNG 1:**
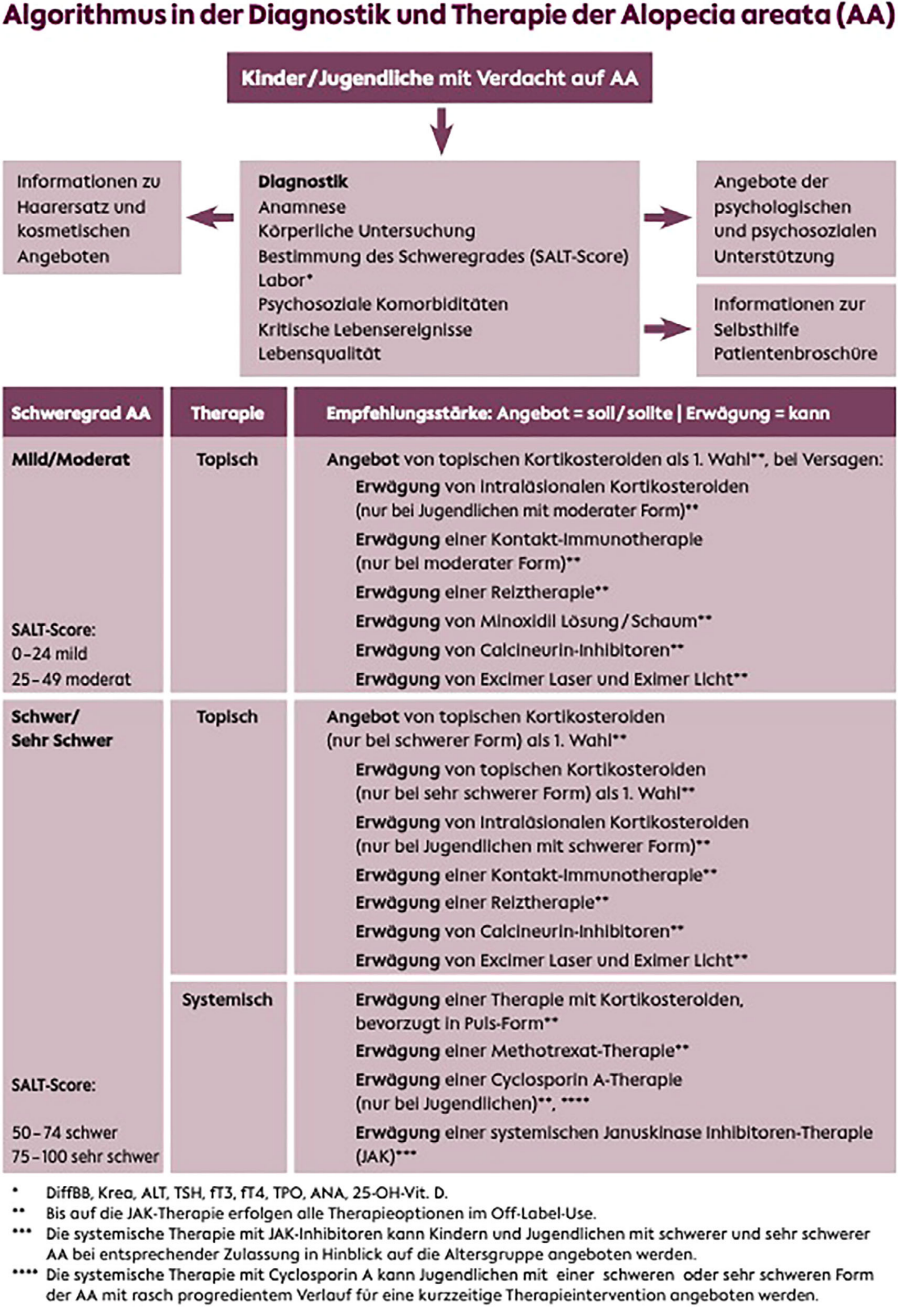
Algorithmus für Kinder und Jugendliche.

**ABBILDUNG 2:**
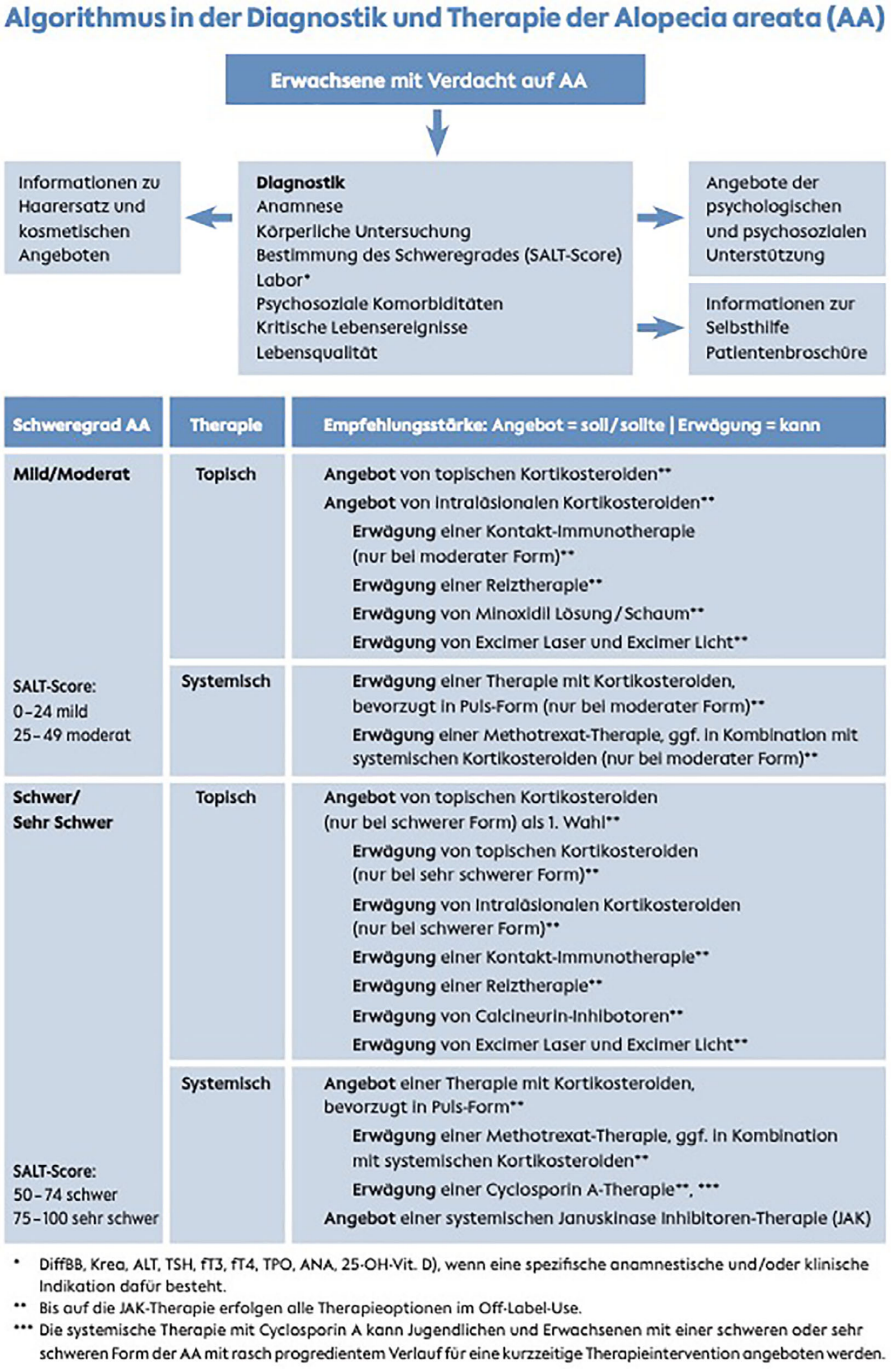
Algorithmus für Erwachsene.

Abbildung 1 zeigt den Algorithmus zur Diagnostik und Therapie für Kinder und Jugendliche. Abbildung 2 fasst die Diagnostik und Therapie für Erwachsene zusammen. Anschließend werden die Empfehlungen zur Diagnostik präsentiert. Die Nummerierung der Empfehlungen entspricht der Nummerierung in der Langversion.

### Anamnese

**Table:** 

	**Empfehlung**	**Empfehlungsstärke**
**E10**	Für die Anamnese *sollen* das Alter bei Erstmanifestation, der Krankheitsverlauf, die aktuelle Krankheitsaktivität einschließlich früherer Schübe, die Dauer der aktuellen und der vergangenen Schübe sowie damit verbundene Symptome erfragt werden.	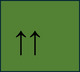
	Starker Konsens	**Konsensbasiert**

**Table:** 

	**Empfehlung**	**Empfehlungsstärke**
**E11**	In der Anamnese *sollen* andere Autoimmun‐ oder Entzündungserkrankungen wie Atopie, Autoimmunkomorbidität (zum Beispiel Schilddrüsenerkrankungen, Vitiligo, entzündliche Darmerkrankungen), rezidivierende Infektionen oder Entzündungsfoci erfragt werden.	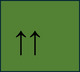
	Starker Konsens	**Konsensbasiert**

**Table:** 

	**Empfehlung**	**Empfehlungsstärke**
**E12**	In der Anamnese *soll* zusätzlich nach einer positiven Familienanamnese einer AA oder anderen Autoimmunerkrankungen gefragt werden.	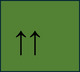
	Starker Konsens	**Konsensbasiert**

**Table:** 

	**Empfehlung**	**Empfehlungsstärke**
**E13**	In der Anamnese *soll* zusätzlich nach früheren Therapien der AA und dem Ansprechen darauf gefragt werden.	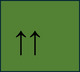
	Starker Konsens	**Konsensbasiert**

**Table:** 

	**Empfehlung**	**Empfehlungsstärke**
**E14**	Ein zeitlicher Zusammenhang von kritischen Lebensereignissen (Life‐Events) mit der Auslösung einer AA *sollte* in der Anamnese erfragt und die Bedeutung kritisch bewertet werden.	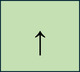
	Starker Konsens	**Konsensbasiert**

Die Erhebung der speziellen Anamnese zur AA umfasst das Alter bei Erstmanifestation zur Stratifizierung in Altersgruppen unterschiedlicher Verlaufsprognosen (zum Beispiel vor oder nach der Pubertät, junge Erwachsene, vor/nach Menopause/Adrenopause), den Krankheitsverlauf (akut, intermittierend, chronisch), aktuelle Krankheitsaktivität, das Erfassen früherer Schübe, die Dauer des aktuellen und der vergangenen Schübe sowie die damit verbundenen Symptome wie Missempfindungen, Juckreiz und andere. Darüber hinaus werden in der Anamnese andere Autoimmun‐ oder Entzündungserkrankungen wie atopische Diathese (Rhinitis allergica, Asthma bronchiale, atopische Dermatitis), Autoimmunkomorbidität (zum Beispiel Schilddrüsenerkrankungen, Vitiligo, entzündliche Darmerkrankungen), rezidivierende Infektionen oder akute und/oder chronische Entzündungsfoci sowie alle aktuellen oder kürzlich durchgeführten topischen oder systemischen Therapien erfragt. Die Erhebung der Familienanamnese in Bezug auf AA und auch Autoimmunerkrankungen wird bei der Erstvorstellung mit erfragt, da für die Beratung sowohl eine positive Familienanamnese für AA als auch für andere Autoimmunerkrankungen relevant sind.^26^ Darüber hinaus sind Angaben zu vergangenen kritischen Lebensereignissen der letzten 3 Monate zu erfragen. Lebensereignisse (*life events*) können als Ereignisse definiert werden, die den meisten Menschen irgendwann im Laufe ihres Lebens widerfahren, die aber dennoch für den Einzelnen ein außergewöhnliches Ereignis darstellen.^27^ Zu den Lebensereignissen gehören Trauerfälle, die Geburt eines Kindes, neuer Job oder Heirat.^28^ Daten aus Befragungen von AA‐Patienten zeigen, dass 24 % der AA‐Patienten von kritischen Lebensereignissen berichteten,^29^ oder im Vergleich zu gesunden Kontrollfällen häufiger vorkommen.^30^, ^31^

### Körperliche Untersuchung

**Table:** 

	**Empfehlung**	**Empfehlungsstärke**
**E15**	Im Rahmen einer körperlichen Untersuchung *soll* eine makroskopische Inspektion der Kopfhaut und des gesamten Integuments, insbesondere der haartragenden Bereiche und der Nägel sowie eine Bestimmung der Ausdehnung und des Haarausfallmusters durchgeführt werden.	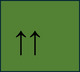
	Starker Konsens	**Konsensbasiert**

**Table:** 

	**Empfehlung**	**Empfehlungsstärke**
**E16**	Im Rahmen einer körperlichen Untersuchung *soll* das Erscheinungsbild der Kopfhaut und die Haut des Integuments innerhalb der haarlosen Areale auf Anzeichen von Narbenbildung, Schuppung, erythematösen Papeln, Pusteln oder Krusten untersucht werden, um andere Differenzialdiagnosen auszuschließen.	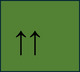
	Starker Konsens	**Konsensbasiert**

Eine sorgfältige klinische Untersuchung beinhaltet eine makroskopische Inspektion der Kopfhaut und des gesamten Integuments, insbesondere der Körperbehaarung und der Nägel sowie eine Bestimmung der Ausdehnung und des Haarausfallmusters. Speziell auch die Behaarung der Arme und Beine wird mit evaluiert. Das Vorgehen der Ganzkörperuntersuchung erfordert einen behutsamen umsichtigen Umgang. Auch wenn das Muster des umschriebenen Haarausfalls einer AA normalerweise charakteristisch ist, wird in selteneren Fällen wie zum Beispiel bei der diffusen Alopecia areata (DAA) oder bei der Alopecia areata incognita (AAI) eine diffuse Verringerung der Haardichte beobachtet. Zusätzlich wird das Erscheinungsbild der Kopfhaut und die Haut innerhalb der haarlosen Areale auf Anzeichen von Narbenbildung, Schuppung, erythematösen Papeln, Pusteln oder Krusten untersucht, um andere Differenzialdiagnosen auszuschließen.^26^

### Diagnostische Methoden

**Table:** 

	**Empfehlung**	**Empfehlungsstärke**
**E17**	Zur Diagnosestellung einer AA *sollte* bei jeder Patientin/jedem Patienten mit Verdacht auf eine AA eine dermatoskopische Untersuchung der alopezischen Areale durchgeführt werden.	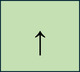
	Starker Konsens	**Konsensbasiert**

**Table:** 

	**Empfehlung**	**Empfehlungsstärke**
**E18**	Zur Verlaufskontrolle einer AA *sollte* eine dermatoskopische Untersuchung der alopezischen Areale durchgeführt werden.	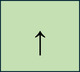
	Starker Konsens	**Konsensbasiert**

### Dermatoskopische Untersuchung

Die dermatoskopische Untersuchung der Kopfhaut, Haarfollikel und Haarschäfte, auch Trichoskopie genannt, ist eine einfache, nichtinvasive mikroskopische Untersuchung, die bei der Diagnosestellung und Verlaufskontrolle von Kopfhaut‐ und Haarerkrankungen sehr hilfreich ist.^32^, ^33^, ^34^ Die dermatoskopische Untersuchung ist besonders wichtig für die Unterscheidung zwischen nicht vernarbenden und vernarbenden Haarerkrankungen, speziell auch für die Bestätigung der klinischen Verdachtsdiagnose einer AA und die Bewertung ihrer Krankheitsaktivität. Generell empfiehlt sich zunächst eine trockene dermatoskopische Untersuchung, um Schuppen oder perifollikuläre Keratosen nicht zu übersehen, gefolgt von Auftragen von Immersionsöl oder Desinfektionsspray, bei Dermatoskopen mit polarisiertem Licht kann darauf verzichtet werden. Die häufigsten dermatoskopischen Befunde bei AA sind gelbe Punkte (*yellow dots*), schwarze Punkte (*black dots*), abgebrochene Haare, Ausrufezeichenhaare und Vellushaare, während andere, weniger häufig beschriebene Befunde senkrechte Haare (*upright hairs*), spitz zulaufende Haare (*tapered hairs*), Schweineschwänzchen‐Haare (*pigtail hairs*) und Pohl‐Pinkus‐Verengungen sind.^35^, ^36^, ^37^, ^38^ Detaillierte Beschreibungen der einzelnen Befunde sind in Tabelle 4 zusammengefasst. Gelbe Punkte treten vor allem bei lang bestehenden Läsionen und bei schwereren Formen der AA, AT und AU auf. Obwohl *yellow dots* ein typischer Befund für eine AA sind, können sie auch bei anderen Haarerkrankungen wie der androgenetischen Alopezie (AGA) oder Trichotillomanie auftreten.^34^ Schwarze Punkte und kurze, abgebrochene Haare sind typische Befunde im aktiven Randbereich von AA‐Herden, beides kann aber auch bei Trichotillomanie beobachtet werden. Ausrufezeichenhaare sind pathognomonisch für die AA und werden typischerweise am aktiven Rand von AA‐Herden gefunden. Vellushaare in typischerweise durch Intermediär‐ oder Terminalhaare charakterisierten Bereichen werden mit einer Remission oder einer lang anhaltenden Erkrankung in Verbindung gebracht.^38^

**TABELLE 4:** Charakteristische dermatoskopische Befunde bei Alopecia areata (modifiziert nach Lintzeri et al. 2022,^26^)

Dermatoskopische Befunde bei AA	Beschreibung
*Yellow dots*	Runde, gelbe oder rosa‐gelbe kreisförmige Punkte, welche die erweiterten, aber intakten Haarfollikelöffnungen darstellen, die mit Talg oder Überresten von Keratinozyten gefüllt sind
*Black dots*	Überreste von im Haarkanal abgebrochenen Haarschäften, vor allem bei dunkelhaarigen Patienten mit hellem Hauttyp
Abgebrochene Haare	Kurze, abgebrochene Haarschäfte
Ausrufezeichen‐Haare	Kurze, abgebrochene Haare, die sich zu ihrem proximalen Ende hin verjüngen
Vellushaare	Dünne, unpigmentierte Flaumhaare
Senkrechte Haare	Gesunde, senkrecht nachwachsende Haare mit einem spitz zulaufenden distalen Ende; auch zu finden bei Telogen‐Effluvium, Trichotillomanie, Tinea capitis und temporaler triangulärer Alopezie
Spitz zulaufende Haare	Normal aussehende Haare mit einem spitz zulaufenden proximalen Ende; Vorläufer von Ausrufezeichenhaaren und schwarzen Punkten
*Pigtail hairs*	Kurze, nachwachsende, zusammengerollte Haare mit spitz zulaufenden Enden; weisen auf einer Remission der Ausfallsepisode hin
Pohl‐Pinkus‐Einschnürungen	Fortschreitende und unregelmäßige Verschmälerung entlang des Haarschafts; Hinweis auf den Schweregrad der Erkrankung; auch bei Chemotherapie‐induzierter Alopezie zu finden

### Haarzupftest

**Table:** 

	**Empfehlung**	**Empfehlungsstärke**
**E19**	Für die Differenzialdiagnose und zur Bestimmung der Krankheitsaktivität zu Beginn und im Verlauf der Erkrankung *sollte* bei jeder Patientin/jedem Patienten mit Verdacht auf eine AA ein Haarzupftest am Rand der Läsionen und der kontralateralen klinisch nicht betroffenen Seite durchgeführt werden.	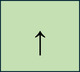
	Starker Konsens	**Konsensbasiert**

Für die Differenzialdiagnose und zur Bestimmung der Krankheitsaktivität ist ein Haarzupftest hilfreich. Ein Büschel von 50–60 Haaren wird kopfhautnah fest gefasst und mäßig in Wuchsrichtung gezogen, und zwar am Rand der einzelnen Läsionen und auf der kontralateralen, klinisch nicht betroffenen Seite.^39^, ^40^ Haarewaschen oder Bürsten der Haare beeinflusst nicht die Aussagekraft des Haarzupftests.^41^ Ein positiver Haarzupftest mit Epilation von ≥ 10 % der erfassten Haare weist auf eine aktive Erkrankung hin, während ein negativer Test mit weniger als zwei ausgezupften Haaren auf ein „normales“ Ausfallen der Haare hinweist.^41^ Ein positiver Zupftest an einer klinisch nicht erkennbar betroffenen Stelle kann auf eine fortschreitende Erkrankung mit diffusem Verlauf hinweisen.^42^

### Trichogramm

**Table:** 

	**Empfehlung**	**Empfehlungsstärke**
**E20**	Ein Trichogramm *kann* in Einzelfällen zur Diagnostik, speziell zur Differenzialdiagnostik durchgeführt werden.	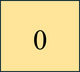
	Starker Konsens	**Konsensbasiert**

Der Einsatz eines Trichogramms in der Diagnostik einer AA wird international kontrovers bewertet. Es handelt sich hierbei um eine mikroskopische Haarwurzeluntersuchung nach Epilation und Einbettung von 60–80 Haaren, welche 5 Tage nach der letzten Haarwäsche mittels Klemme und geübter Zupftechnik entnommen werden.

Rudnicka und Kollegen halten das Trichogramm für ein hilfreiches ergänzendes Zusatz‐Instrument für die klinische Bewertung, die Diagnose und die Überwachung des Ansprechens auf die Behandlung.^33^ Eine andere Expertenrunde bewertet das Trichogramm als nicht hilfreich für die Diagnostik. Uneinig waren sich die Experten auch in der Bewertung des Trichogramms zur Beurteilung der Krankheitsaktivität.^32^ Das Trichogramm könne allenfalls eine DAA von einem Telogeneffluvium unterscheiden, da eine DAA durch das Vorhandensein von anagen‐dysplastischen und dystrophen Haaren gekennzeichnet ist.^43^ Zu bedenken ist allerdings, dass ein Überwiegen von Telogenhaaren durch die entzündliche Invasion von Haarfollikeln mit vermehrter Induktion der Telogenphase auch im chronischen Stadium einer AA beobachtet werden kann.

### Biopsie

**Table:** 

	**Empfehlung**	**Empfehlungsstärke**
**E21**	Eine Biopsie der Kopfhaut *sollte* bei Verdacht auf eine diffuse AA oder bei nicht eindeutiger Klinik zum Ausschluss anderer Differentialdiagnosen durchgeführt werden.	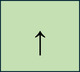
	Starker Konsens	**Konsensbasiert**

**Table:** 

	**Empfehlung**	**Empfehlungsstärke**
**E22**	Wenn für die Diagnosestellung einer AA eine Biopsie erfolgen soll, *soll* diese am Rand des umschriebenen Herdes unter Vermeidung einer für die androgenetische Alopezie typischen Stelle durchgeführt werden.	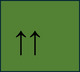
	Starker Konsens	**Konsensbasiert**

In einer internationalen Expertenabstimmung bewerteten alle Beteiligten übereinstimmend, dass bei einem nicht eindeutigen klinischen Befund einer AA in ausgewählten Situationen Hautbiopsien in Betracht gezogen werden können, um andere Erkrankungen auszuschließen.^33^

Eine Kopfhautbiopsie ist insbesondere dann angezeigt, wenn eine vernarbende Alopezie klinisch nicht sicher ausgeschlossen werden kann, eine einzelne Läsion behandlungsresistent ist oder bei DAA zur differenzialdiagnostischen Abklärung.^32^ Im Zusammenhang mit einer AA ist eine einzige Biopsie in der Regel ausreichend, um die Diagnose zu stellen. Die Biopsie wird am Rand des umschriebenen Herdes durchgeführt unter Vermeidung einer für androgenetische Alopezie typischen Stelle.^32^ Eine zusätzliche Kopfhautbiopsie aus nicht‐läsionaler Kopfhaut wird als nicht wichtig erachtet.^32^ Die histopathologischen Befunde bei AA hängen von der Aktivität der Krankheit zum Zeitpunkt der Biopsie ab. Im akuten (frühen) Stadium ist das Hauptmerkmal im Befund ein peribulbäres und intrabulbäres lymphozytäres Infiltrat, das die anagenen oder katagenen Follikel umgibt und als „Bienenschwarm“ beschrieben wird. Das Infiltrat besteht hauptsächlich aus CD4^+^ und CD8^+^ T‐Zellen; es können jedoch auch eosinophile Zellen, Mastzellen und Plasmazellen nachgewiesen werden. Zusätzlich wird ein verfrühter oder prozentual erhöhter Übergang in die katagene oder telogene Phase beobachtet. Bei langjährig persistierender (chronischer) AA kann die Intensität der Infiltration variieren; die meisten HF befinden sich in der telogenen Phase, und es können auch miniaturisierte HF vorhanden sein. Es kann aber auch eine erhöhte Anzahl leerer HF beobachtet werden. Teilweise zeigen Keratinpfröpfe in leeren Follikelostien einen langanhaltenden Verlauf ohne Nachwachsen an.^44^, ^45^

### Differenzialdiagnose

**Table:** 

	**Empfehlung**	**Empfehlungsstärke**
**E23**	Bei klinisch nicht eindeutigem Befund einer AA *soll* in Abhängigkeit vom klinischen Befund und der Altersgruppe eine sorgfältige Differenzialdiagnostik erfolgen.	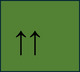
	Starker Konsens	**Konsensbasiert**

Die Differenzialdiagnose (DD) einer AA umfasst vor allem Erkrankungen, die mit nicht vernarbendem, umschriebenem oder diffusem Haarausfall einhergehen. Abhängig von der Altersgruppe müssen unterschiedliche DD in Betracht gezogen werden: Trichotillomanie, Tinea capitis und temporale trianguläre Alopezie sind die wichtigsten Krankheitsbilder, die bei der Unterscheidung von umschriebener AA vor allem im Kindes‐ und Jugendalter in Betracht gezogen werden sollten, während ein Telogeneffluvium, eine AGA mit weiblichem Haarausfallmuster und medikamenteninduzierte Alopezie hauptsächlich bei diffusen Formen von AA für die DD in Betracht gezogen werden. Das gesamte Spektrum der DD der AA ist in Tabelle 5 zusammengefasst.^26^

**TABELLE 5:** Häufige Differenzialdiagnosen der umschriebenen und diffusen Alopecia areata, gruppiert nach Altersgruppen (Kinder/Jugendliche und Erwachsene).

	DD umschriebene AA	DD diffuse AA
Kinder/Jugendliche	Tinea capitis	loses oder kurzes Anagenhaar Syndrom
	Trichotillomanie	Telogenes Effluvium
	Temporale trianguläre Alopezie (Naevus Brauer)	Kongenitale Hypotrichose
Jugendliche/Erwachsene	Follikuläre Muzinosen, follikulotrope Mycosis fungoides	Telogenes Effluvium
	Alopecia syphilitica	AGA mit weiblichem Haarausfallmuster
	Vernarbende Alopezie wie chronisch diskoider Lupus erythematodes (CDLE), Lichen planopilaris	Medikamenteninduzierte (antiproliferativ) Alopezie

*Abk*.: AA, Alopecia areata; DD, Differenzialdiagnose; AGA, androgenetische Alopezie

### Klassifizierungsinstrumente

**Table:** 

	**Empfehlung**	**Empfehlungsstärke**
**E24**	Zur Bestimmung des Schweregrades der AA bei Erstvorstellung und zur Verlaufsbeurteilung *sollte* der SALT‐Score verwendet werden.	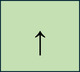
	Starker Konsens	**Konsensbasiert**

**Table:** 

	**Empfehlung**	**Empfehlungsstärke**
**E25**	Zur Bestimmung des Schweregrades der Beteiligung der Augenbrauen, der Wimpern und der Nägel *kann* der Einsatz des ClinRo‐Instruments oder von PRO‐Skalen für Wimpern und Augenbrauen erwogen werden.	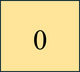
	Starker Konsens	**Konsensbasiert**

Die Bewertung des Schweregrades der AA ist für die Beratung und Therapieplanung der Patienten von großer Bedeutung. Die Ausprägung einer AA leitet den Arzt bei der therapeutischen Entscheidungsfindung, und hilft, das Ansprechen auf die Therapie abzuschätzen und die Prognose der Krankheit einzuschätzen. Um die Bewertung des Ausmaßes und des Verlaufs der AA in klinischen Studien zu erleichtern und zu standardisieren, wurde ein einfacher, aber zuverlässiger und reproduzierbarer Schweregrad‐Score entwickelt: der *Severity of Alopecia Tool (SALT)‐Score*.^46^ Der SALT‐Score wird in der klinischen Praxis als Maß für die Einschätzung der Ausdehnung der AA im Bereich der Kopfhaut verwendet.^32^ Hierbei wird die Kopfhaut in vier Quadranten unterteilt, die jeweils einen Prozentsatz (%) der gesamten Kopfhautfläche darstellen: linke Seite (18 %), rechte Seite (18 %), Oberseite (40 %) und Rückseite (24 %). Um den SALT‐Score, der ein Maximum von 100 % hat, zu ermitteln, wird der Prozentsatz des Haarverlustes in jedem Quadranten visuell geschätzt und dann addiert. Anhand des so berechneten SALT‐Scores lassen sich fünf Schweregrade des Haarverlusts unterscheiden: S0 = kein Haarverlust, S1 < 25 % Haarverlust, S2 = 25–49 % Haarverlust, S3 = 50–74 % Haarverlust, S4 = 75–99 % Haarverlust, S5 = 100 % Haarverlust.^46^, ^47^ Üblicherweise wird ein SALT ≥ 50 als schwere AA und ein SALT≥75 als sehr schwere AA angesehen. Eine Limitation des SALT‐Scores ist darin zu sehen, dass er die mögliche Beteiligung anderer anatomischer Bereiche nicht berücksichtigt, die aber für die Beurteilung der Schwere der Erkrankung und der Prognose wichtig sind. Um auch die Manifestationen der AA außerhalb der Kopfhaut zu berücksichtigen, stehen weitere Bewertungsskalen zur Verfügung, die den SALT‐Score ergänzen. So können Augenbrauen‐, Wimpern‐, Bart‐, Achselhöhlen‐, Schamhaar‐ und andere Beteiligungen bewertet werden, um die Charakterisierung individueller Patienten zu verbessern.^48^ Diese Methode berücksichtigt dabei das Ausmaß der Kopfhautbeteiligung (SALT‐Score), das Muster der Kopfhaut‐AA, die Anzahl anderer betroffener anatomischer Stellen und ihre Ausdehnung. In ähnlicher Weise entwickelten Jang et al. 2016 den *Alopecia Areata Progression Index (AAPI)* zur Bewertung der Gesamtaktivität des Haarausfalls bei AA‐Patienten mit pigmentiertem Haar, indem sie zum SALT‐Score klinische Befunde im Zusammenhang mit Haarausfall (Zupf‐Test und dermatoskopische Untersuchungsbefunde) hinzufügten und so ein erweiterter Score berechnet werden kann.^49^ Zur Einschätzung des Schweregrades der Beteiligung der Augenbrauen, Wimpern und der Nägel sind Instrumente wie das ClinRo‐Instrument und andere patientenorientierte Einschätzungsinstrumente (PRO) verfügbar.^50^ Idealerweise wird die Einschätzung durch eine Fotodokumentation begleitet.^50^

Zusammenfassend lässt sich sagen, dass in der täglichen klinischen Praxis die Berechnung des SALT‐Scores, die Identifizierung anderer betroffener anatomischer Stellen und die Inspektion der Nägel auf AA‐bedingte Läsionen wesentliche Elemente bei der Beurteilung der Krankheitsschwere sind.

### Laborwerte

**Table:** 

	**Empfehlung**	**Empfehlungsstärke**
**E26**	Bei Vorliegen einer AA *sollte* ein Screening auf eine Schilddrüsenerkrankung durchgeführt werden.	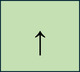
	Starker Konsens	**Konsensbasiert**

**Table:** 

	**Empfehlung**	**Empfehlungsstärke**
**E27**	Bei Erwachsenen mit AA *sollten* Blutuntersuchungen (Differenzialblutbild, Nieren‐ und Leberfunktion), Schilddrüsenwerte (TSH, TPO, ± TRAK, ± TG), ANA‐Titer und 25‐OH‐Vitamin D durchgeführt werden, wenn eine spezifische anamnestische und/oder klinische Indikation dafür besteht.	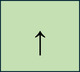
	Starker Konsens	**Konsensbasiert**

**Table:** 

	**Empfehlung**	**Empfehlungsstärke**
**E28**	Bei Kindern und Jugendlichen mit AA *sollten* zum Screening auf mögliche Komorbidität Blutuntersuchungen (Differenzialblutbild, Nieren‐ und Leberfunktion), Schilddrüsenwerte (TSH, fT3, fT4, TPO), ANA‐Titer und 25‐OH‐Vitamin D untersucht werden.	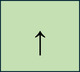
	Konsens	**Konsensbasiert**

**Table:** 

	**Empfehlung**	**Empfehlungsstärke**
**E29**	Bei anamnestischem und/oder klinischem Hinweis auf das Vorliegen von weiteren Autoimmunerkrankungen *sollte* eine gezielte Labordiagnostik erfolgen.	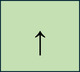
	Starker Konsens	**Konsensbasiert**

**Table:** 

	**Empfehlung**	**Empfehlungsstärke**
**E30**	Eine infektionsserologische Untersuchung, insbesondere hinsichtlich viraler Auslöser, sofern es anamnetisch und klinisch orientierend keinen zusätzlichen Infektionsverdacht gibt, *soll nicht* bestimmt werden.	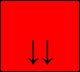
	Starker Konsens	**Konsensbasiert**

**Table:** 

	**Empfehlung**	**Empfehlungsstärke**
**E31**	Vor der Einleitung einer systemischen Behandlung der AA *sollen* die gleichen (Labor)‐Untersuchungen für das einzusetzende Medikament wie bei anderen dermatologischen Erkrankungen entsprechend Fachinformation und allgemeinen Handlungsempfehlungen des jeweiligen Wirkstoffs durchgeführt werden.	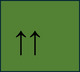
	Starker Konsens	**Konsensbasiert**

Es gibt bisher keine validierten Biomarker, die bei der Diagnose einer AA bestimmt werden können.^33^ Zu etlichen Laborparametern herrscht international Uneinigkeit, so beispielsweise ob routinemäßige Screenings auf Vitamin‐D‐Mangel und Schilddrüsenerkrankungen durchgeführt werden sollten.^32^ Dennoch entschied sich die Leitliniengruppe für die oben formulierte Empfehlung. Darüber hinaus beschränken sich folgende Untersuchungen nur auf den Einzelfall, sie sind nicht bei allen Patienten mit der Diagnose AA und vor allem nur zur differenzialdiagnostischen Abklärung empfohlen: vollständiges Blutbild, Nieren‐ und Leberfunktion, Screening auf weitere Autoimmunerkrankungen, Bindegewebserkrankungen, Zöliakie, perniziöse Anämie und Diabetes.

In Ermangelung relevanter klinischer Symptome und Anzeichen ist eine infektionsserologische Untersuchung auf Viren nicht sinnvoll, um einen potenziellen Auslöser für eine AA‐Episode zu identifizieren. Der morgendliche Cortisolspiegel ist kein nützlicher Test bei Patienten, die glauben, dass Stress eine AA‐Episode ausgelöst haben könnte. Vor der Einleitung einer systemischen Behandlung der AA sind die gleichen Untersuchungen erforderlich wie bei Anwendung der jeweiligen Therapeutika bei anderen dermatologischen Erkrankungen.^32^

### Mykologie

**Table:** 

	**Empfehlung**	**Empfehlungsstärke**
**E32**	Eine mykologische Abklärung *soll* durchgeführt werden, wenn ein klinischer Verdacht auf Tinea capitis besteht.	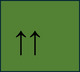
	Starker Konsens	**Konsensbasiert**

Eine mykologische Diagnostik stellt keine routinemäßige Untersuchung dar. Sie ist nur erforderlich, wenn ein klinischer Verdacht auf Tinea capitis besteht.

### Komorbidität und assoziierte Autoimmunerkrankungen

**Table:** 

	**Empfehlung**	**Empfehlungsstärke**
**E33**	In der Diagnostik *sollte* auf psychosoziale Komorbidität, vor allem Angst (soziale Angst) und Depression sowie Stigmatisierung geachtet werden.	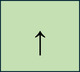
	Starker Konsens	**Konsensbasiert**

Die AA kann zusammen mit verschiedenen anderen Erkrankungen auftreten. Tabelle 6 fasst die Beschreibung der Häufigkeiten der Komorbidität zusammen:

**TABELLE 6:** Häufigkeiten von Komorbidität bei Alopecia areata.

Komorbidität	Häufigkeit	Referenz
Vitamin‐D‐Mangel^1^	65,4 % (95 %‐KI 50,6–77,8)^*^	^51^
Helicobacter‐pylori‐Infektionen^1^	62,8 % (95 %‐KI 46,2–76,9)^*^	
Psychiatrische Erkrankungen^1^	49,2 % (95 %‐KI 17,8–81,5)^*^	
Angst^1^	27,1 % (95 %‐KI 17,7–39,2)^*^	
Depression^1^	18,9 % (95 %‐KI10,9–30,8)^*^	
Atopische Erkrankungen^1^	20,6 % (95 %‐KI 16,2–25,9)^*^	
Allergische Rhinitis^1^	17,7 % (95 %‐KI 14,1–21,9)^*^	
Atopische Dermatitis^1^	9,6 % (95 %‐KI 6,2–14,4)^*^	
Schilddrüsenerkrankungen^1^	8,0 % (95 %‐KI 5,9–10,7)^*^	
Hyperinsulinämie^2^	60,8 % (95 %‐KI 46,9–73,1)^*^	
Alexithymie^2^	52,9 % (95 %‐KI 37,1–68,2)^*^	
Metabolisches Syndrom^2^	37,3 % (95 %‐KI 25,2–51,2)^*^	
Linsenveränderungen^2^	32,1 % (95 %‐KI 18,7–57,8)^*^	
Retinale Veränderungen^2^	24,0 % (95 %‐KI 13,8–38,5)^*^	
Audiologische Veränderungen^2^	17,3 % (95 %‐KI 0,5–89,4)^*^	
Atopische Dermatitis	RR 2,9; 95 %‐KI 2,7–3,2^**^	^5^
Pruritus	RR 2,7; 95 %‐KI 2,4–3,1^**^	
Lupus erythematodes	RR 2,4; 95 %‐KI 1,7–3^**^	
Urtikaria	RR 2,3; 95 %‐KI 1,9–2,7^**^	
Angstzustände	8 %^***^	^52^
atopische Dermatitis	5 %^***^	
Autoimmunthyreosen	4 %^***^	

*Abk*.: AA, Alopecia areata; KI, Konfidenzintervall; RR, Relatives Risiko

^1^ Ergebnisse basieren auf mehreren Studien mit insgesamt hohen Fallzahlen.

^2^ Ergebnisse basieren auf sehr kleinen Fallzahlen aus wenigen Studien.

* Prävalenz basiert auf mindestens einer Studie mit einer Vergleichsgruppe mit Gesunden, bezogen auf AA‐Erkrankte.

** Bei einer Prävalenz < 3 % bezogen auf die gesamte Bevölkerung

*** Prävalenz bezogen auf AA‐Erkrankte mit Komorbidität

## PROGNOSTISCHE FAKTOREN

Prognostische Faktoren sagen den zukünftigen Verlauf einer Erkrankung vorher.^53^ Wir bezeichnen an dieser Stelle alle Faktoren, die den weiteren Krankheitsverlauf, die Ausprägung von AA sowie das Therapieansprechen beeinflussen können, als prognostische Faktoren. Diese sind für das Verständnis und die Planung des Behandlungskonzeptes sowie für die Patientenberatung besonders wichtig.

Die ausführliche Beschreibung der Literatur erfolgte in der Langversion der Leitlinie, hier werden lediglich die durch die Literaturrecherche identifizierten prognostischen Faktoren und deren Bedeutung in den Tabellen 7 und 8 gezeigt.

**TABELLE 7:** Bedeutung als prognostischer Faktor.

Endpunkt	Prognostischer Faktor	Studienergebnisse/Expertenmeinung
Progressiver Verlauf	Positive Eigenanamnese für Autoimmunerkrankungen	Expertenmeinung
	Positive Eigenanamnese für Atopie	Expertenmeinung
	Dauer der Erkrankung über fünf Jahre	Expertenmeinung
	Ophiasis‐Typ	Expertenmeinung
	Nagelbeteiligung	Expertenmeinung
	Verlust der Wimpern und der restlichen Körperbehaarung	Expertenmeinung
Ansprechen der Therapie	Alter bei Erstmanifestation	Expertenmeinung
	Autoimmun‐ oder atopische Erkrankung in der Anamnese	Studienergebnisse^54^, ^55^, ^56^, ^57^, ^58^, ^59^, ^60^
	Nagelbeteiligung^*^	Studienergebnisse^57^
Rückfall	Familienanamnese einer AA	Studienergebnisse^61^
	Erkrankungsdauer > 6 Monate	Studienergebnisse^54^, ^62^

* Nagelbeteiligung: widersprüchliche Ergebnisse, daher in Tabellen 7 und 8 aufgeführt

*Abk*.: AA, Alopecia areata

**TABELLE 8:** Keine Bedeutung als prognostischer Faktor.

Endpunkt	Prognostischer Faktor	Studienergebnisse/Expertenmeinung
Ansprechen der Therapie	Eisenmangel	Expertenmeinung
	Impfung	Expertenmeinung
	Hypothyroidismus (Kinder)	Studienergebnisse^56^
	Atopische Dermatitis (Kinder)	Studienergebnisse^56^
	Atopische Diathese (Kinder)	Studienergebnisse^56^
	Erkrankungsdauer > 1 Jahr	Studienergebnisse^57^
	Nagelbeteiligung^*^	Studienergebnisse^54^, ^58^, ^60^
	Zeitpunkt der Erstmanifestation	Studienergebnisse^54^, ^55^, ^63^
	Familienanamnese einer AA	Studienergebnisse^54^, ^55^, ^56^, ^58^, ^59^, ^60^, ^61^, ^64^
Progressiver Verlauf	Positive Familienanamnese für eine organspezifische Autoimmun‐ oder atopische Erkrankung	Expertenmeinung
	Einleitung einer Therapie der AA in den ersten sechs Monaten	Expertenmeinung

* Nagelbeteiligung: widersprüchliche Ergebnisse, daher in Tabellen 7 und 8 aufgeführt

## LEBENSQUALITÄT ZUM ZEITPUNKT DER DIAGNOSE

Die Auswirkungen von Haarausfall können für jeden einzelnen Betroffenen weit über rein optische Belange hinausgehen,^65^ was häufig psychologische und psychosoziale Folgen haben kann. Dazu gehören ein vermindertes Gefühl der persönlichen Attraktivität, ein geringeres Selbstvertrauen und Selbstwertgefühl sowie negative Auswirkungen auf soziale Interaktionen.^66^ Die Analyse der Lebensqualität von Menschen mit AA zeigt, wie sie ihre körperliche, geistige und soziale Gesundheit im Zusammenhang mit ihrer Krankheit wahrnehmen.^67^ Der chronische oder häufig schubweise Verlauf der AA trägt dabei erheblich zur Beeinträchtigung der Lebensqualität der Patienten bei.

Zur Erfassung der Lebensqualität stehen diverse Instrumente zur Verfügung, von generischen Instrumenten bis hin zu dermatologisch spezifischen Instrumenten wie der *Dermatology Quality of Life Index* (DLQI).^68^ Die Gesamtwerte des DLQI können wie folgt interpretiert werden: 0–1 = keine Auswirkung auf das Leben des Patienten, 2–5 = geringe Auswirkung, 6–10 = moderate Auswirkung, 11–20 = sehr große Auswirkung, 21–30 extrem große Auswirkung auf das Leben des Patienten.^68^

In einer Metaanalyse wurden aus 14 Studien Daten des DLQI‐Scores von 3978 Erwachsenen mit einer AA und einem durchschnittlichen Alter von 23 bis 51 Jahren zusammengeführt. Der gepoolte Wert des DLQI lag bei 6,67 (95 %‐KI 5,54–7,81). Dieser Wert wird als moderate Beeinträchtigung der Lebensqualität gewertet. Die Autoren der Metaanalyse stellten bei den eingeschlossenen Studien eine hohe Heterogenität in den Ergebnissen fest, die durchschnittlichen DLQI‐Werte reichten von 2,1 (95 %‐KI 1,91–2,29) bis 10,6 (95 %‐KI 9,53–11,85). Die Metaanalyse lässt offen, inwiefern der Schweregrad der AA, die Art der Therapie oder die Dauer der Erkrankung die Lebensqualität beeinflussten.^69^

Daten einer amerikanischen Querschnittstudie zeigten jedoch Zusammenhänge mit dem Schweregrad der AA, dem Verlust der Augenbrauen und der Lebensqualität auf. In der Studie wurden Daten von 259 Patienten analysiert, die eine leichte Form der AA hatten (durchschnittlicher SALT‐Score 12,8). Als Instrument zur Messung der Lebensqualität wurde der Skindex‐16‐Fragebogen genutzt. Es zeigte sich eine durchschnittliche Lebensqualität von 37,1 (SD 23,6) Punkten bei einem möglichen Maximalwert von 100 Punkten, ein höherer Wert bedeutet eine höhere Beeinträchtigung der Lebensqualität. Die Autoren berechneten per Regressionsanalyse zusätzlich Faktoren, die eine Beeinträchtigung in der Lebensqualität vorhersagen. Das Ergebnis zeigte, dass der Schweregrad der AA, eine Augenirritation und der Verlust der Augenbrauen eine höhere Beeinträchtigung in der Lebensqualität vorhersagten (*moderate severity*: 12,9; 95 %‐KI 6,1–19,6); *severe severity*: 14,9; 95 %‐KI 6,7–23,2; *eye irritation*: 37,0; 95 %‐KI 7,1–66,9; *eyebrow hair loss*: 6,8; 95 %‐KI –0,5–14,0, wobei das Ergebnis für den Faktor der Augenirritation mit großer Unsicherheit verbunden ist, nur zwei Patienten in der Studie haben eine Augenirritation angegeben. Die Dauer der Erkrankung hatte keinen prädiktiven Wert (0,8; 95 %‐KI 0,3–1,3), und bei Männern zeigte sich, dass sie eher eine höhere Lebensqualität hatten im Vergleich zu Frauen (–10,7; 95 %‐KI –16,0 bis –5,4).^70^

## DANKSAGUNG

Open access Veröffentlichung ermöglicht und organisiert durch Projekt DEAL.

## INTERESSENKONFLIKT

Die vollständige Liste der erklärten Interessenkonflikte ist im Leitlinienreport unter https://register.awmf.org/de/leitlinien/detail/013‐104 zu finden.
